# Supplementary material for: Uncovering the phonon spectra and lattice dynamics of plastically deformable InSe van der Waals crystals
Source: Nat Commun. 2024 Jul 24;15:6248. doi: 10.1038/s41467-024-50249-5 (PMC11269642; doi:10.1038/s41467-024-50249-5)
Supplement: Supplementary file 1 — Supplementary Information [file 41467_2024_50249_MOESM1_ESM.pdf]

Supplementary Information: Uncovering the phonon spectra and lattice dynamics of plastically deformable InSe van der Waals crystals

Jiangtao Wu[1, 13], Yifei Lin[2, 13], Mingfang Shu[1, 13], Yifei Liu[3], Yupeng Ma[3], Gaoting Lin[1], Cuiping Zhang[1], Pengfei Jiao[1], Fengfeng Zhu[4], Yan Wu[5], Russell A. Ewings[6], Helen C. Walker[6], Guochu Deng[7], Songxue Chi[5], Shengwei Jiang[1], Matteo Baggioli[8], Min Jin[9], Haozhe Wang[10], Weiwei Xie[10], Tian-Ran Wei[3, *], Jiong Yang[2, *], Xun Shi[3,11], and Jie Ma[1,12, *]

[1]*Key Laboratory of Artificial Structures and Quantum Control, School of Physics and Astronomy, Shanghai Jiao Tong University, Shanghai 200240, China*
[2]*Materials Genome Institute, Shanghai University, 99 Shangda Road, 200444 Shanghai, China*
[3]*State Key Laboratory of Metal Matrix Composites, School of Materials Science and Engineering, Shanghai Jiao Tong University, Shanghai 200240, China.*
[4]*State Key Laboratory of Functional Materials for Informatics, Shanghai Institute of Microsystem and Information Technology, Chinese Academy of Sciences, 200050 Shanghai, China*
[5]*Neutron Scattering Division, Oak Ridge National Laboratory, Oak Ridge, Tennessee 37831, United States*
[6]*ISIS Pulsed Neutron and Muon Source, STFC Rutherford Appleton Laboratory, Harwell Campus, Didcot, OX11 0QX, United Kingdom*
[7]*Australian Centre for Neutron Scattering, Australian Nuclear Science and Technology Organisation, Lucas Heights, NSW, Australia*
[8]*Wilczek Quantum Center and School of Physics and Astronomy, Shanghai Jiao Tong University, Shanghai 200240, China*
[9]*College of Materials, Shanghai Dianji University, Shanghai 201306, China*
[10] *Department of Chemistry, Michigan State University, East Lansing, Michigan 48824, United States*
[11]*State Key Laboratory of High Performance Ceramics and Superfine Microstructure, Shanghai Institute of Ceramics, Chinese Academy of Sciences, Shanghai 200050, China*
[12]*Collaborative Innovation Center of Advanced Microstructures, 210093, Nanjing, Jiangsu, China*

[13]These authors contributed equally: Jiangtao Wu, Yifei Lin, Mingfang Shu

[*]Corresponding authors: tianran_wei@sjtu.edu.cn (T.-R. Wei); jiongy@t.shu.edu.cn (J. Yang); jma3@sjtu.edu.cn (J. Ma)

I. Structure and slip analysis

Supplementary Figure 1 shows the measured and calculated Bragg reflection intensities of InSe specimen based on single-crystal XRD. The integration of the data using the β-phase structure (hexagonal, P6(3)/mmc) yielded a total of 1880 reflections to a maximum θ angle of 36.41° (0.60 Å resolution), of which 480 were independent (average redundancy 3.917, completeness = 96.5%, R_{int} = 10.91%, R_{sig} = 8.68%) and 402 (83.75%) were greater than $2\sigma(F^2)$. By contrast, the R_{int} value for the ε phase model is ~25%. Therefore, the synthesized InSe can be indexed to the β phase.

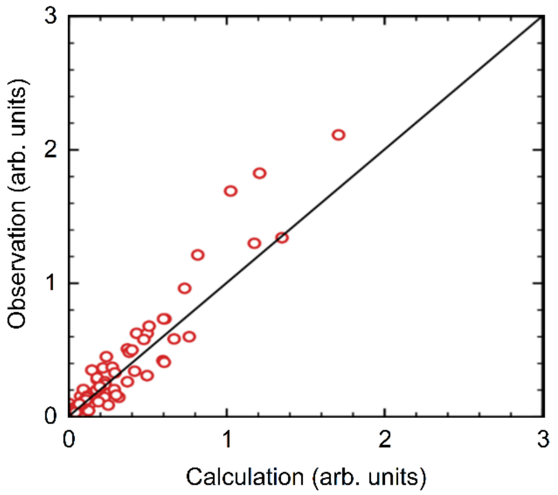

Supplementary Figure 1. Comparison of the observed Bragg reflection intensities at 300 K on the single-crystal XRD and the simulated ones based on the β-phase structure. The solid line is a guide to the eye.

Supplementary Figure 2 shows the Raman spectra at 300 K. Three main peaks are observed, at about 114.6, 176.1 and 226 cm^{-1}, corresponding to the $A_1(\Gamma_1^2)$, $E(\Gamma_3^3)$/ $E(\Gamma_3^1)$-(TO) and $A_1(\Gamma_1^3)$ vibrational modes.[1] However, for non-centrosymmetric 3R phase InSe, another vibrational mode $A_1(\Gamma_1^1)$-LO should appear at 199 cm^{-1}.[2] In combination with elastic neutron scattering and SAED data, the specimens in this work are indexed to the β-phase.

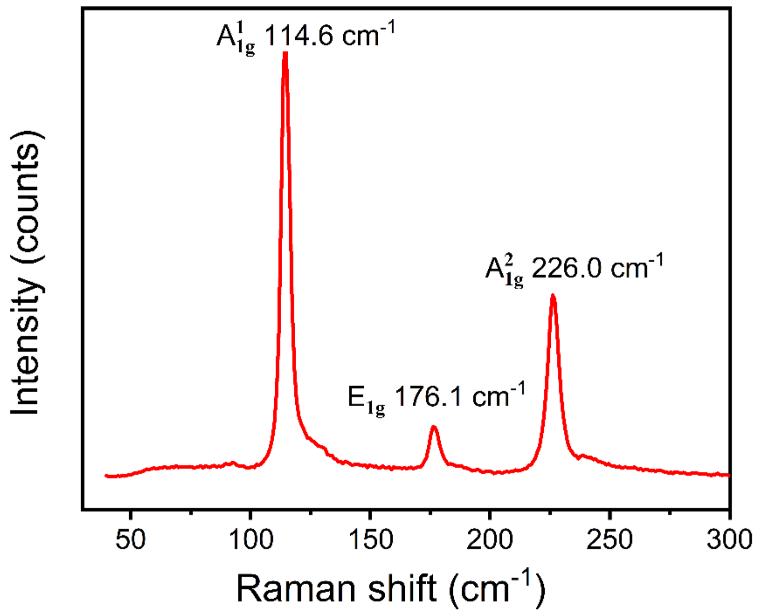

Supplementary Figure 2 Raman spectrum of InSe at room temperature.

We briefly illustrate the *ab* plane of InSe. To simplify the analysis, only directions of reciprocal vectors are labeled (***a****, ***b****, ***α****, ***α'****, ***β**** and ***β'****, where prime for axis after interlayer slip) while the magnitudes are ignored. In the structure of InSe, where the angle between *a*- and *b*-axis is 120 degrees, the orthogonalization is a mere 2-dimensional problem as illustrated in Fig.S3. The interlayer slip along [K-K0] direction is equivalent to along ***α****, leading to a tilt angle *θ* between ***c*** and ***c'*** (***c*** for origin axis, ***c'*** for axis after slip). According to the relation: $\boldsymbol{\alpha}^* = \frac{2\pi}{V}\boldsymbol{\beta} \times \boldsymbol{c}$, $\boldsymbol{\alpha}'^* = \frac{2\pi}{V}\boldsymbol{\beta} \times \boldsymbol{c}'$, the angle between ***α**** and ***α'**** is *θ*. That is, there is a tilt angle *θ* between the original [K-K0] direction and the slipped [K-K0] direction, while ***c**** (= ***c'****) should be maintained due to the constant *α*, *β* axis. Based on the geometric relation mentioned above, we can illustrate the reflections in (K-KL) before and after slip. Compared with the experiment result (panel below the schematic), the schematic agrees with the experimental situation. Furthermore, we can obtain the slip distance： $\Delta\ell = c^*\tan\theta =$

$$c\,\frac{0.75 \times [00\mathrm{L}]\ \mathrm{unit}}{1 \times [\mathrm{K{-}K0}]\mathrm{unit}} = 16.68 \times \frac{0.75 \times 0.37723}{1.822} = 2.58\ \text{Å}$$

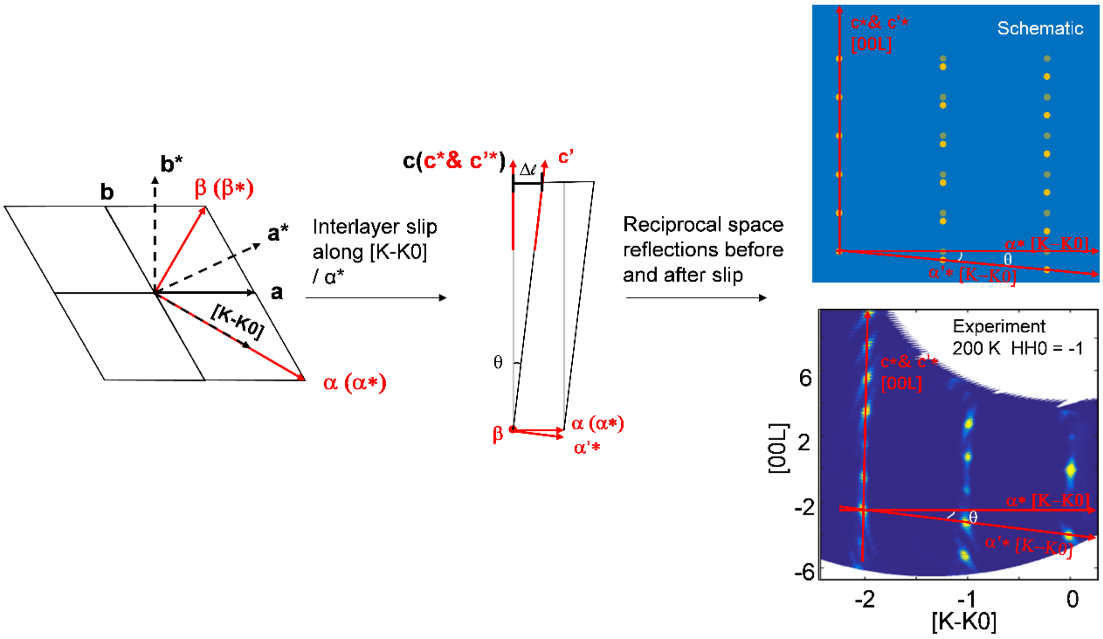

Supplementary Figure 3 Schematics of interlayer slip process and geometric calculation.

II. Theoretical calculation and inelastic neutron scattering

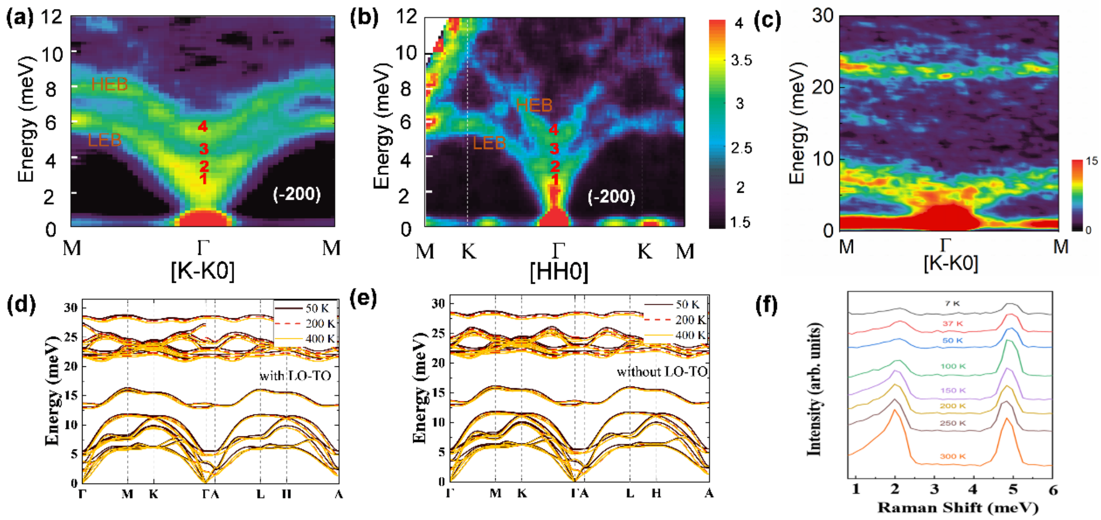

Supplementary Figure 4 The phonon dispersions observed in the dynamical susceptibility $\chi''(q, \mathrm{E})$ from (a),(b) inelastic neutron scattering (INS) measurements. (c) The phonon spectra of whole energy scale along [K-K0] at 50 K. (d), (e) Temperature dependent phonon dispersion by AIMD simulations (with and without LO-TO splitting). (f) Temperature-dependence of Raman spectra experimentally.

To investigate the temperature effect on the phonon dispersions, *ab initio*

molecular dynamics simulations were conducted as shown in Supplementary Figure 4 (d). The phonon softening behaviors are obvious at the zone center. Especially, the lowest energy TO mode shows more temperature dependence, which indicates that the interlayer shear mode is more anharmonic and the associated crystal structure is more unstable. The temperature-dependence of the Raman spectra were presented in Supplementary Figure 4 (f). The softening behavior of high-energy band (HEB) phonon, ~ 5 meV, and the low-energy band (LEB) phonon, ~2meV, are both clearly observed in the AIMD results. As the nesting of acoustic-optical modes enables a large number of three-phonon scattering channels and amplifies the effect of anharmonicity in the potential, the AIMD simulation on second-order phonon interaction only partially presented this anharmonic effect.

Supplementary Table 1. The AIMD-calculated phonon frequencies, symmetries, and Raman/IR activeness of the Γ-point phonons for β-InSe at 200 K.

β-InSe AIMD 200 K							
meV<br>cm^{-1}	2.03<br>16.4	3.78<br>30.5	4.71<br>38.0	5.01<br>40.4	12.90<br>104.0	13.70<br>110.5	21.66<br>174.7
Irre. Rep.	E_{2g}^1<br>① in Fig.2f	B_{2g}^1<br>② in Fig.2f	E_{2u}^1<br>③ in Fig.2f	E_{1g}^1<br>④ in Fig.2f	B_{1u}^1	A_{1g}^1	E_{1g}^2
Raman active	√	-	-	√	-	√	√
meV<br>cm^{-1}	21.75<br>175.4	22.73<br>183.3	22.90<br>187.7	24.06<br>194.0	24.41<br>196.9	28.49<br>229.8	28.53<br>230.1
Irre. Rep.	E_{2u}^2	E_{2g}^2	E_{1u}	A_{2u}	B_{2g}^2	B_{1u}^2	A_{1g}^2
Raman active	-	√	-	-	-	-	√

In Supplementary Table 1, we labeled the symmetries and energies of all the optical phonons at Γ-point for β-InSe at 200 K. The modes with Raman / infrared active are also labeled in the table. Compared with the results in Ref. [3], Supplementary Table 2, the symmetries are the same, and the phonon frequencies are slightly different due to the different exchange-correlation functionals (R^2SCAN metaGGA in the work vs. LDA), lattice parameters, and the methods to compute force constants (AIMD in this work vs. DFPT). Furthermore, the Raman active modes are

also consistent with the experimental measurements in this work, except for the E_{2g} mode. The missing of the E_{2g} mode in the Raman measurement of β-InSe was previously discussed. [4]

Inelastic neutron scattering (INS) measurements were conducted with the instrument MERLIN, at the ISIS Neutron and Muon Source. The sample was aligned with the (HHL) plane horizontal. The measurements were performed at 50 K and 200 K with incident energy E_i=22 meV, respectively. The corresponding energy resolution (full width at half maximum) is 0.8meV at elastic line. The four-dimensional scattering function, $S(q, E)$, where q is the momentum transfer, was obtained after data collection and reduction. The imaginary dynamical susceptibility, $\chi''(q, E) = S(q, E)/(n(E)+1)$, where $n(E)$ is Bose distribution, was sliced along particular high symmetry directions to provide two-dimensional views, as shown in Fig. 2 (a) and (b) of the main text.

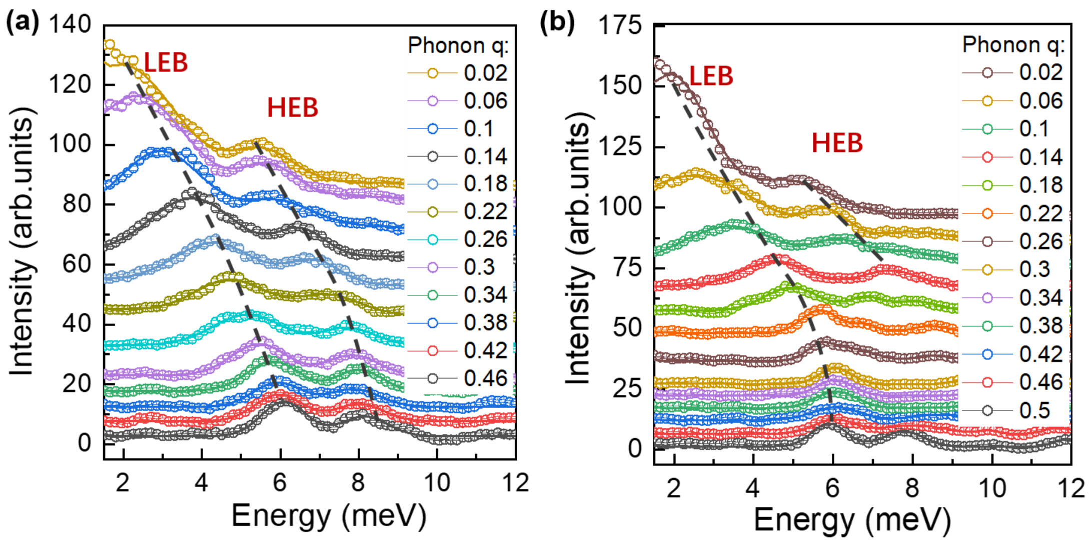

Supplementary Figure 5 (a) and (b) Constant-q cuts for q along [K-K0] and [HH0] in (-200) zone from MERLIN data at 200 K (E_i = 22 meV), respectively. The dashed lines are guides for the eye, indicating the LEB and HEB phonon excitations.

To investigate the LEB and HEB phonons in the (-200) zone, constant-q cuts from the MERLIN data in Fig.2 of main text were constructed. The integral range of intensity was $\Delta q_{[K\text{-}K0]}$ = 0.04 rlu at each step ($\Delta q_{[HH0]}$ = 0.1 rlu, $\Delta q_{[00L]}$ = 0.1 rlu) for

Supplementary Figure 5(a) while it was $\Delta q_{[HH0]}$ = 0.04 rlu at each step ($\Delta q_{[K\text{-}K0]}$ = 0.1 rlu, $\Delta q_{[00L]}$ = 0.1 rlu) for Supplementary Figure 5(b). The figures show the intensity vs positive energy with the correction of detailed balance. Two phonon bands are clearly observed, labeled as LEB and HEB, respectively. The LEB is broad at low *q*, getting sharper near the zone boundary. While the HEB shows a weak *q* dependence of phonon linewidths in the whole BZ. The peak position and linewidth were fitted with a Gaussian function. The full-width at half maximum of the Lorentzian fit was taken to obtain the phonon linewidth with the deconvolution of instrument resolution by the software *tobyfit*.

It is clear that the linewidth of the LEB is even larger than its position near the BZ center. Moreover, the linewidths of phonon branches in InSe are larger at the center of the BZ than at boundaries. All these features indicate giant anharmonicity of the LEB near the zone center.

Compared to LEB, HEB is very complicated with the contributions of LA, LO and TO. The energies and linewidths of HEB were fitted by *tobyfit*. The HEB phonon dispersions are not temperature dependent and flatter than the phonons of LEB with small group velocity, meanwhile, the phonon linewidths of HEB are smaller than the values of LEB. Hence, the lattice thermal conductivity of HEB is small.

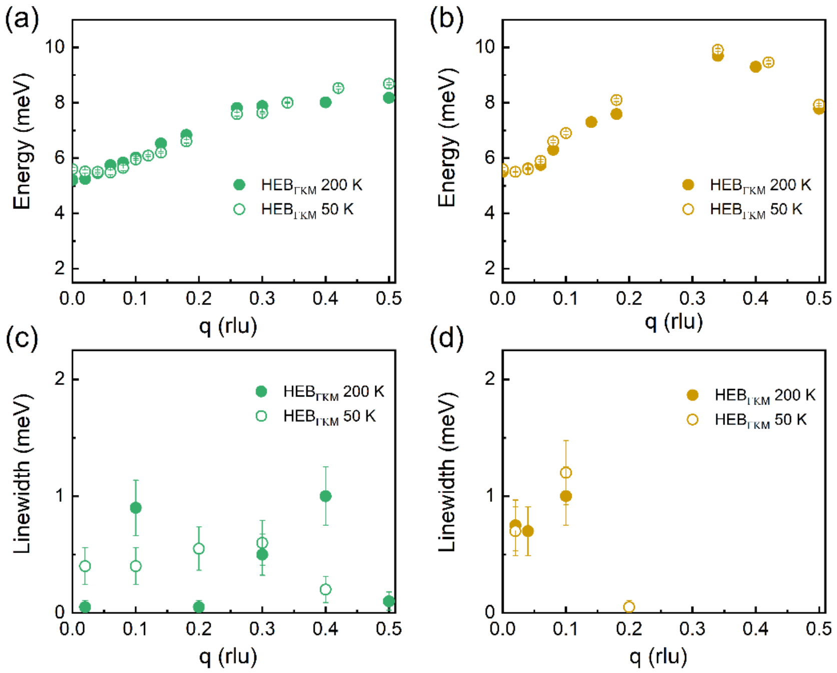

Supplementary Figure 6 The fitted HEB phonon dispersions and linewidths along ΓM (a, c)

and ΓKM (b, d).

III. Heat capacity and thermal conductivity analysis

To investigate the thermal dynamics in details, the C_p data are fitted by considering the contribution of both Debye and Einstein vibrations:[5,6]

$$C_p(T) = C_D[9k_B\left(\frac{T}{\theta_D}\right)^3 \int_0^{x_D} \frac{x^4 e^x}{(e^x-1)^2}dx] + \sum_i C_D[3R\left(\frac{\theta_{E_i}}{T}\right)^2 \frac{e^{\frac{\theta_{E_i}}{T}}}{\left(e^{\frac{\theta_{E_i}}{T}}-1\right)^2}], \quad (1)$$

where R is the universal gas constant, k_B is the Boltzmann constant, θ_D and θ_{Ei} are the Debye and Einstein temperatures.

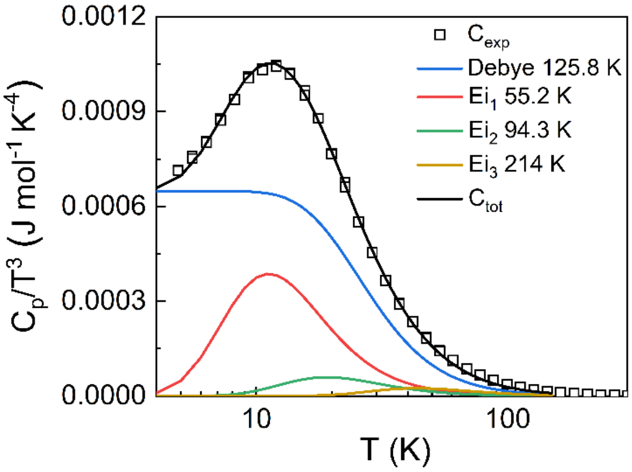

Supplementary Figure 7 The Debye representation Cp/T^3 of InSe from measurement (hollow black squares). A fitting was applied by a Debye model (blue curve) superimposed to three Einstein models (red, olive and dark yellow curves, respectively).

As the unit cell is expressed as In_4Se_4 with 3 acoustic modes and 21 optical modes for a 3D system, one Debye term and three Einstein terms are applied to fit the data. The fitted Debye temperature is 125.8 K, consistent with the value of 128 K determined by sound velocity.[7] The three Einstein modes are 55.2 K for θ_{Ei1} (4.75 meV), 94.3 K for θ_{Ei2} (8.12 meV) and 214 K for θ_{Ei3} (18.44 meV). Typically, the low Einstein modes will lead to strong acoustic-optical phonon interaction, which will reduce the phonon lifetime as well as group velocity, leading to low heat conduction.

The C_p data are also fitted by considering the contribution of one Debye mode, C_D, and three Einstein modes, C_1, C_2, C_3, respectively. The fitting parameters are

shown in Supplementary Table 2. The fitted Debye temperature is consistent with the value determined by sound velocity.

Supplementary Table 2 fitting parameters of heat capacity of InSe

**Fitting parameters**	**InSe**
C_D	0.66 (±0.006)
θ_D (K)	125.8 (±0.14)
C_1	0.12 (±0.002)
θ_{E1} (K)	55.2 (±0.31)
C_2	0.09 (±0.002)
θ_{E2} (K)	94.3 (±2.56)
C_3	0.45 (±0.002)
θ_{E3} (K)	214.5 (±8.19)
R^2	0.9986
Reduced-χ^2	1.4E-10

The presence of excess heat capacity, the so-called boson peak, is common for some amorphous solids, which deviate from the Debye T^3 law at low temperature. Here, we show the glassy feature of crystalline InSe in Supplementary Figure 7. To investigate the origin of the anomaly, we obtained the phonon density of state (DOS) from AIMD simulation at 200 K, as well as the harmonic approximation (labeled as DFT) based on the finite displacement approach, respectively. The contributions of phonons to the heat capacity can be computed from the phonon DOS $g(E)$, as:[8]

$$S_{ph} = 3k_B \int dEg(E)[(n+1)\ln(n+1) - nln\, n], \quad (1)$$

where $n(T, E)$ is the Bose-Einstein distribution for temperature T and phonon energy E, $n = [\exp\left(\frac{E}{k_B T}\right) - 1]^{-1}$. The phonon DOS was obtained by AIMD simulation. Therefore, the contribution of harmonic phonons, $C_{ph,H}$, can be derived from Eq. (1), referring to the relation: $C_{ph,H} = T dS_{ph} / dT$. In addition to the total phonon DOS, we also calculated the partial DOS as the projection of each phonon branch. Based on the

partial DOS, the contribution of each branch to the heat capacity was obtained. As shown in Supplementary Figure 8 (a) for acoustic modes and (b) for optical modes, the disappearance of ZA mode gives the high consistence of experimental results, which indicates the ZA acoustic phonon branch may be significantly affected by the disorder in the system.

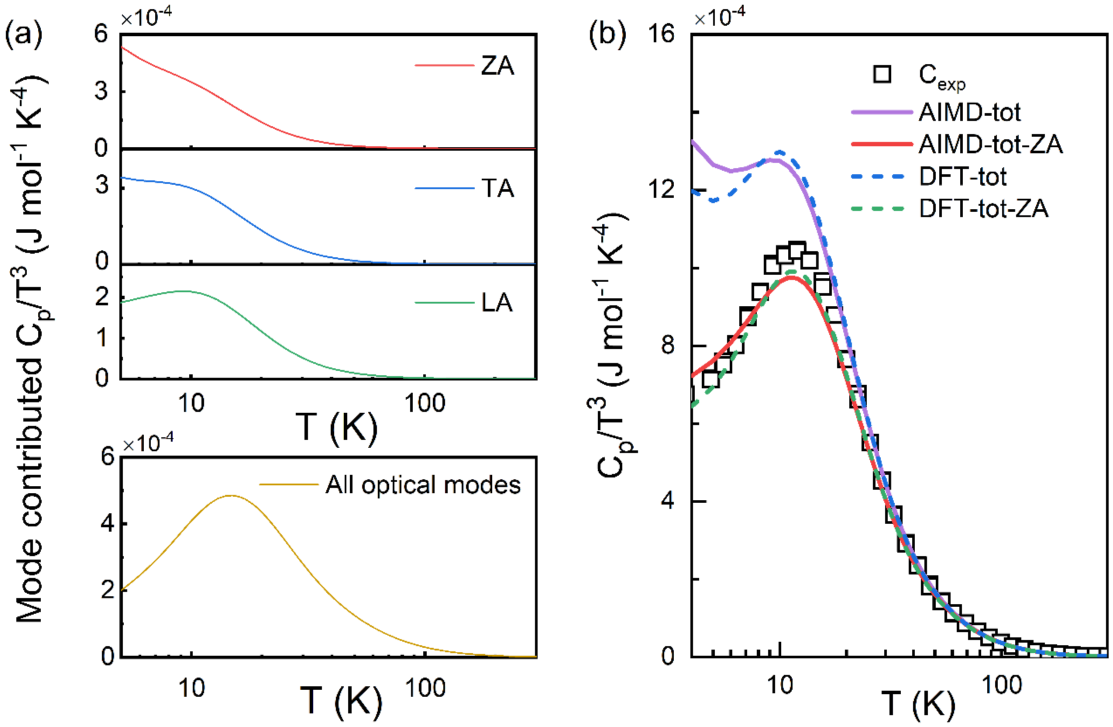

Supplementary Figure 8 (a) The calculated mode-contributed Cp/T^3 for the three acoustic modes (ZA, TA and LA) and for all optical modes; (b) The Cp/T^3 data include curves from both AIMD and first-principles simulations (DFT, standard harmonic calculation at 0 K): the light purple and red curves are generated from AIMD, with the light purple considering all phonon DOS and the red excluding the ZA contribution. The blue and green dashed curves are from first-principles simulations, with the blue dashed line considering all phonon DOS and the green dashed line excluding the ZA contribution.

Supplementary References

1 Grimaldi, I. *et al.* Structural investigation of InSe layered semiconductors. *Solid State Communications* **311**, 113855 (2020). https://doi.org:10.1016/j.ssc.2020.113855

2 Dai, M. J. *et al.* Properties, synthesis, and device applications of 2D layered InSe. *Advanced Materials Technologies* **7**, 2200321 (2022) *https://doi.org:10.1002/admt.202200321*

3 Bejani, M. *et al.* Electronic structure, vibrational properties, and optical spectra of two- and three-dimensional hexagonal InSe: Layer-dependent ab initio calculations. *Physical Review Materials* **6**, 115201 (2022). https://doi.org/10.1103/PhysRevMaterials.6.115201

4 Carlone, C. *et al.* Optical Phonons and Crystalline Symmetry of InSe. *physica status solidi (b)* **103**, 123-130 (1981). https://doi.org:10.1002/pssb.2221030112

5 Kittel, C. *Introduction to solid state physics*. 8 edn, (John Wiley & Sons, 2004).

6 Dimitrov, I. K. *et al.* Einstein modes in the phonon density of states of the single-filled skutterudite $Yb_{0.2}Co_4Sb_{12}$. *Physical Review B* **82**, 174301 (2010). https://doi.org/10.1103/PhysRevB.82.174301

7 Shi, H. *et al.* Dynamic carrier transports and low thermal conductivity in n-type layered InSe thermoelectrics. *Aggregate* **2**, e92 (2021). https://doi.org:10.1002/agt2.92

8 Wallace, D. C. *Statistical Physics of Crystals and Liquids*. (World Scientific, 2003).
